# Reply to Dr. Patterson of Londonderry, on the Subject of Chorea Sancti Viti

**Published:** 1807-09

**Authors:** John Redman Coxe


### *Reply to* Dr. PATTERSON *of Londonderry, on the Subject of* CHOREA SANCTI VITI. By JOHN REDMAN COXE, M. D.

IN the 15th volume of the London Medical and Physical Journal, Dr. Patterson of Londonderry, has published an answer to a communication of mine in a preceding volume of that work, extracted from the 8th volume of the New-York Medical Repository; entitled, "Observations on Chorea Sancti Viti, with a new Theory of the Disease;" in which the Doctor accuses me of having censured not only some of his reasoning, but also part of his practice, "in a manner at once abrupt, rough, and magisterial." Conscious of having never intended to give the slightest offence to Dr. P. in that essay, (of the truth of which I request every gentleman to convince himself by a candid perusal of the piece) I should not now have answered his illiberal and unjust aspersions, had he not called upon me "for refutal or concession."

It seems extraordinary, that such harsh epithets should be applied to me, because I ventured to differ in opinion from my learned opponent, by saying, "it appears to me, that Dr. Patterson has greatly erred, in supposing chorea the primary disease; of which each must judge for himself." I see nothing "abrupt, rough, or magisterial," in thus allowing every one to form his own opinion of the subject in dispute, after comparing the arguments on both sides. Surely I have the same right to my sentiments that the Doctor has to his: and he will find it difficult to persuade mankind that *his ipse dixit* is to carry conviction to the breast of every reader; or, that an individual may not be allowed the privilege of dissenting from him, without being presumed to act from unworthy motives!

The Doctor supposes I have censured his practice, by saying, (in referring to the case he relates in his letters concerning the internal dropsy of the brain,) "the operation of the emetics probably hastened her dissolution, by determining too forcibly the circulation to the brain," (an expression he seems to sneer at,) "and hence exciting an acute state of hydrocephalus."

Whilst I still adhere to the same opinion, justice impels me to absolve myself from any idea of imputing to the medical attendant any blame, from the administration of the emetics. I had not read the Doctor's remarks with so little attention, as not to have noticed that their exhibition was not his fault. The manner of expressing myself, might to a candid mind have confirmed this; for (although Dr. P. appears to be unacquainted with that urbanity which should characterize medical men in their opposition to each other's sentiments, yet, knowing how liable we all are to mistakes,) had the Doctor been a principal on that occasion, I should have been peculiarly careful of speaking in a manner to wound either his feelings or his reputation. I should not, so virulently as he has done, have exclaimed,

"Quot Themison ægros autumno occiderit uno."

Considered, however, in a practical point of view, I must oppose, as far as an individual can, the use of emetics in those complaints, especially when increased action of the vessels exists; the practice may be sanctioned by "eminent professional characters."

"Nullius jurare in verba magistri."

Even several of the gentlemen he has named, are guarded in their expressions, by the terms, *gentle* and *mildest*, in recommending the use of emetics.*

The

* That the Doctor does not himself approve of the use of emetics, I think we may fairly deduce from his own words, although he has ventured in his answer to quote the authority of several medical men in their favour. "If Dr. Quin" (says Dr. P. p. 6. of his letters,) "had felt even a faint conception of the present theory, would he in this case have prescribed, early in the disease," and if not early, why in its most advanced state? a remarkably strong vomit of ipecacuanha and antimonial wine?"—And in speaking of errhines, (p. 52.) he says, that before using them, "precautionary considerations are very necessary; our subjects are tender, irritable, and affected with plethora of the head, the particular part on which these medicines operate, and to which they give such a degree of commotion, as would, *no doubt*, prove highly pernicious in the incipient stage of the disease. Nay, I am not clear, that they are perfectly safe in the more advanced periods, when vascular repletion may be diminished, and serous effusion on the approach, or in any degree existing. For I apprehend, that the shock of sneezing, as well as the *agitation* of *vomiting*, if *by chance* eventually *harmless*, would not promote absorption in the advanced torpid state, and would be a vexatious experiment to the patient, to whom the smallest motion of the head is generally so great an annoyance,"

In speaking of the administration of cathartics, he says, (p. 34.) "For a stimulating purgative must either be in such quantity, or of such quality, as would in many cases, especially where the stomach is so frequently disordered, prove actually emetic; an effect which you, (Dr. Quin) *properly observe*, would *hasten considerably the progress of the disease*, and I would add, an effect which must be highly pernicious at its commencement." He afterwards says, (p. 35.) "Touching vomits we may here remark, that several years ago, it was started as a question, whether they be, or be not, safe medicines in febrile disorders, *caput petentes?* Applying

The Doctor thinks it behoved me, before pronouncing that he had greatly erred, &c. to refute the reasoning on which his conclusion is founded; at which, however, he says, I have not made the smallest attempt, "the performance of which is so necessary to vindicate my dogmatism."—Whilst, however, a doubt remains on my mind, whether the Doctor comprehends his own reasoning, if such it can be termed; I should recommend his looking at home for a plentiful crop of dogmas dispersed throughout all his writings. His "innate nervous stimulus," and "principle of irritability," which he so confidently tells us, "is not confined to the fibre, or simple solid; but is also possessed by the fluids of the body, particularly by the blood;" I conceive to belong to this class; unless indeed, the Doctor has better reasons to confirm them, than mere assertion. His assertion, that the heart "*must be* the centre of the principle," I regard as equally unqualified and devoid of proof. Of its "influence on the contents of the cranium," I desire no other proof than the effect of the emetics in the case alluded to.

The Dr. says, "As in those ages, in which this constitution prevails, convulsions likewise prevail, the increased action which they produce will augment the impetus of the circulation, especially in the vessels of the head; which we see is actually the case, since a considerable determination to that part is observed in those disorders. This determination increases the action of the blood-vessels, and inflammation is the consequence. This consequence is forwarded by the plethora, particularly of the head, which exists in the early and advancing stages of life; and the whole train of symptoms constituting *febricula hydrocephalica*, (the name which I appropriate to the disease) is then brought into view.*

Q 2 I perplying the question to hydrocephalus internus, which I reckon of a febrile nature, and most certainly *caput petens;* I would answer, that here emetics *are in my opinion injudicious remedies*, especially in strong doses at the beginning of the malady. And I ask, would you give them at this period, or indeed *at any other*, upon the principle of promoting absorption, by the general concussions arising from their operation?"

* It may here be observed, that in the history which Dr. Patterson gives us of this disorder, (hydrocephalus) after enumerating the symptoms of he first and second stage, he ends those of the third stage, (p. 11) by "impaired deglutition, and *convulsions*, which *form the catastrophe*." I certainly might with as much reason say, that these final convulsions of life, were the cause of the effusion of water, which dissection shews in the

I perfectly agree with him in the effect which is produced by the determination to the head, of inflammation, and of the whole train of symptoms, constituting febricula hydrocephalica, (as Dr. P. expresses it;) but I view the convulsions, which he supposes the *cause* of the increased action, as the *effect* of the plethora, or effusion induced; for that hydrocephalus, induced by inflammation of the brain, should, by pressure on the nerves, (or their origin,) induce convulsions, (especially as it is in a part incapable of distention) is surely a more rational view of the subject, than that the convulsions should excite the determination to the brain, (which he only asks to be conceded as the petitio principii,) and the consequent febricula hydrocephalica. I would only ask, which is the most simple of these doctrines, or whether all the Doctor's reasoning on the subject amounts even to a presumptive proof of it? The Doctor assigns not even a probable cause for the convulsions; but I have, I think, rationally proved the legitimacy of my deduction; and might add, that every case of injury to the brain, from blows, falls, and the like, tends to establish it; inasmuch, as in those instances where death takes place after convulsions, dissection has evinced the cause to be matter formed, or water effused, or great engorgement of the vessels of the brain.

"But how are we to explain (says the Doctor) the occurrence of effusion, where the accumulation of the irritable principle is not so vigorous, and yet the action of stimuli is assiduous? In these cases *I would conjecture*, that the nervous stimulus, acting, *as I suppose*, on a mass of

---

the ventricles, as the Dr. has for asserting chorea, (which he afterwards admits to be merely a *species* of convulsions,) to be its origin. I think too, it may not be irrelative to state, that in the view of the different kinds of inflammation which the Doctor has taken to aid him in the investigation of the subject; he adds, p. 19, " No douht there is a propriety, and even an advantage in distinguishing with accuracy, the organ immediately affected by inflammation; yet it is equally certain, that, in all such cases, the *great diversity* in the symptoms is *more imputable to a difference in the function of the part engaged*, than to any specific variation in the nature of the inflammation." I think, although this is applied to the effect of inflammation, it is not asking too much of the Doctor, to impute the same diversity of symptoms, (that is, the various forms of convulsions,) to a difference in the function of the part engaged, by the greater or less pressure of a fluid, or turgid vessels in the brain, on one part more than on another, at different times. For we cannot but admit that pressure from the same source will produce a different effect on one part of the brain, from what it does on another.

of irritability less moveable than in convulsive habits, excites a passion, which, if violent, suddenly destroys irritability, or the vital principle, and death ensues; or which, if moderate, engages only a part of the irritability, and the natural state of temper is regained for a time." And he illustrates this (reasoning!) with an account of the dissection of Mirabeau, who was found to have "un petit épanchement dans *le cerveau;*"—adding, does not this instance shew, that an irritable temper, almost constantly exercised, may be a cause of effusions in different cavities, and of a watery effusion in the brain; which conditions, *in all probability,* were preceded by more or less inflammation in the diaphanous membranes of those parts? And must we not admit an irregularity of temper in those persons peculiarly subject to hydrocephalus internus?"

I am sorry to say, I see nothing like reasoning in the above conjectures and suppositions. What does the example of Mirabeau prove? What, if an irritable temper almost constantly exercised, is a cause of effusion in different cavities; and even if preceded by inflammation in the diaphanous membranes of those parts; it does not prove convulsions, (unless convulsion and irritable temper are synonimous,) to have excited it. Mirabeau was not subject to these; although, it is probable, had he lived, the increasing effusion might have induced them. Neither can I see the necessity of admitting an irritability of temper, in those persons peculiarly subject to hydrocephalus internus, more than in any other species of dropsy. I would, however, recommend it to the Doctor, (if his theory is just,) to guard against that temperament so conspicuous in his writings, lest it should be followed by the same fatal effect as in Mirabeau.*

I thought I had been sufficiently explicit in attempting to explain, how a small additional effusion, in a case of chronic hydrocephalus, might render it similar to a case of acute; for it is certain, that if the brain is capable of containing by slow increments, ℔j of fluid, without a fatal issue; a small addition to this, suddenly made, though

* It might not have been amiss for the Doctor to have added the small residue at the end of his *reasoning*, as it contains more sense than all the preceding. "To promote this end, namely, the extension of practical knowledge, an accumulation of facts is, undoubtedly the aptest means." If the Doctor could have viewed my cases, as a small addition to the facts elsewhere enumerated, he might have spared his ungenerous remarks.

only to the amount of a tea-spoonful, might nevertheless induce all the symptoms of an acute attack, and even death itself; for the brain being incapable of distention, must have time afforded it to accommodate itself to the effect of pressure, by absorption of a part. There is in this nothing incomprehensible, nothing obscure; and I must still declare my firm belief, that the Doctor's patient fell a victim to an attack of acute hydrocephalus, induced by the operation of the emetics. At least, if a greater effusion of water did not take place suddenly, the dissection shews such an increased determination to the blood vessels of the brain as was fully adequate to the final catastrophe.

The Doctor proceeds to say, that "the influence of convulsive affections in causing accumulations of the fluids in the brain is not a new point in pathology; it has occupied the attention of physicians during some years past. A child was indisposed about 2 months with frequent head-ach, which was supposed to proceed from worms, but anthelmintic medicines afforded no relief, and he died in a convulsive fit. On opening the head, the vessels of the brain were observed to be uncommonly turgid, and in the ventricles was found more than double the ordinary quantity of serum." 'In this case, (says the late Dr. Percival, of Manchester,) I apprehend the turgescence of the vessels was the *effect*, and not the *cause* of the convulsions; for the reflux of the blood from the head to the heart being obstructed during the fit, in which I believe the patient expired, the vascular distention must have been permament. The redness and even the blackness of the face, which takes place in convulsions, affords sufficient proof of sanguineous accumulation;' "hence it appears, that the same speculative opinion, which Dr. Coxe styles a *new theory*, with respect to convulsive motions in hydrocephalus, was conceived at least 13 years before Dr. Coxe wrote, and had obtained so much credit as to induce the learned Dr. Percival to counteract its extension."

Although the attention of physicians during some years, has been occupied by reviewing "the influence of convulsive affections in causing accumulations of the fluids in the brain;" it by no means follows that they were correct in their conjectures. On the contrary, I think every fact tends to shew that, (as Dr. P. has done) they have mistaken the cause for the effect; a circumstance by no means uncommon.

The case of the child mentioned above, is clearly in point. The head-ach for two months might have led to a more more rational deduction than that it proceeded from worms; and the uncommon turgescence of the vessels of the brain, after such a long continued head-ach, might have, I apprehend, led Dr. Percival to regard the convulsive fit of which he died, as the effect of such turgescence; and I doubt not, *to be* in no wise singular in this opinion, although it is opposed to the sentiments of that truly eminent physician. I must say I think Dr. Patterson strives hard to give my opinions to another, when he asserts that Dr. P. had thought it necessary to counteract its extension at least 13 years before I wrote; I see no author of that time, quoted as bringing forward the doctrine; nor any credit which it had then obtained, to induce Dr. P. to counteract its extension. Had the Doctor really conceived it 13 years before; or had it acquired any credit, the extension of which he might think it necessary to counteract; is it probable he would have said so little upon it? And is it not still less probable that Dr. Patterson in his express treatise on the subject, and with this case before him, should have passed it in silence, and not till now, when he thinks it will answer his purpose, detect a theory in Dr. Percival's writings, which any man of common candour will allow to have no pretensions to it? If even I was to admit the fact as such, Dr. Percival is stating a case of convulsions, to which he had not approximated chorea, (the disease I have particularly treated of, as symptomatic of the affection of the brain; and, until Dr. Patterson shews that Dr. P. intended his ideas to extend to chorea, *then* viewed as a primary disease, he must excuse me for still upholding my claim to the new theory, as he (contemptuously) says I style it.

" If, as Dr. Coxe supposes," says Dr. Patterson," chorea be not an idiopathic disease, but arises *solely* as a symptom of the *chronic* hydrocephalus, the characteristic phenomena of the latter should always precede, or accompany, the appearance of the former. Whereas, upon a careful examination, we shall find, that the symptoms of chorea exist without any token of hydrocephalus, as strikingly exemplified in the case of my patient, already alluded to; in whom its essential features prevailed, long before it wrought the change which terminated in the fatal effusion into the ventricles of the brain." The case above alluded to, examined attentively, will, I think, most satisfactorily prove that hydrocephalus is a cause, not an effect, of convulsions. The brain by long and slow effusion, (as was the case with his patient) is enabled to accommodate itself to a pressure, which produced more rapidly, would induce death; witness the numerous cases related by authors. It is strange that Dr. P. should be so attached to the doctrine he holds, as to overlook the objections against it, and to bring forward cases strongly opposing it! I should be disposed to rest the merit of the question on this case, and doubt not that it will convey conviction to the minds of many, who perhaps at present, receive, without reflection, the Doctor's assertions for proof.

"Had the cases of chorea, (says Dr. Patterson) which came under the notice of Sydenham, been accompanied with hydrocephalic symptoms, so careful an observer certainly would have mentioned such striking occurrences, and would not have found as he did, those cases liable to periodical returns, which rarely, or never, happen in any species of hydrocephalus."

Had Sydenham been acquainted with the *acute* state of hydrocephalus, I think there is little doubt, that so sagacious an observer would have drawn an accurate contrast between it and the chronic state; and might also, from the knowledge it would have impressed upon his mind, have been led to investigate more thoroughly the sources of chorea, on which he has so slightly treated. The reason is evident why Dr. Sydenham did not detect the symtoms of hydrocephalus; because in the chronic state, many are wanting (owing to the effect produced by habit, as I have before attempted to explain) which are almost essential to its acute attack. The wonder is less, that Sydenham should have failed in demonstrating the cause of a disease he regarded as primary; when Dr. Patterson, with the advantages he possesses over that illustrous character, and with cases and dissections so strong in point, came no nearer than putting cause for effect.

"Non omnia possumus omnes."

And here I must advert to Dr. P's note, in which I am charged with oversight, in respect to Sydenham's theory of chorea, because I say, he "scarcely adverts to any thing but its curious gesticulations." I confess I did not think it necessary to attempt a confutation of his theory, which ascribes it to an humour thrown on the nerves, &c. I had not overlooked it; but I must leave it for Dr. P. to combat, unless indeed he means to uphold it; which mentioning it as he does, seems to imply. It i indeed, rather surprising, that Dr. P. has not detected my theory theory in Dr. Sydenham's; as mine depends upon the pressure of a fluid on the brain and nerves.

As to my being aware of the difficulty on this point, (Sydenham's, &c. above,) and putting the question, "If hydrocephalus is the cause of chorea, it may be asked, as the former is not an unfrequent disease, why the latter does not more often occur?" I confessed myself ignorant of the reason, but it by no means follows, as the Doctor so confidently affirms, "that it would *always* be produced, if it possessed the affinity of a symptom." I will only ask, if any disorder exists, in which any individual symptom of it, which may be named, is not occasionally absent? Does not the Doctor himself allow, as well as others, that one of the most leading symptoms of hydrocephalus, *viz.* strabismus, is not always present? Cold applied to various parts, will produce a diversity of complaints, attended with their peculiar symptoms: in like manner it may be imagined that pressure on different portions of the brain, may induce a diversity of symptoms. The objection of the Doctor to a thing devoid of certainty, does not prove, either that he is right, or that I am wrong. I must however, be permitted to enjoy my belief, that although hydrocephalus is not always, or even frequently, accompanied by chorea, yet wherever chorea does exist, there hydrocephalus, or its preceding state of inflammation and turgescence of the vessels, (which I presume may also be chronic) also exists as the cause. It is not necessary I apprehend to its existence, that a gallon of water should be effused; for the disease may be as complete with but a tea-spoonful as with a larger quantity; although it may be less dangerous, inasmuch as the brain will sooner accommodate itself to the pressure.

The Doctor asserts, that in his second case of chorea, not a symptom of hydrocephalus can be traced, and adds, that my second case is of the same description. To this I may observe, that the assertion by no means proves that water did not exist in the brain. The Doctor, however, gives reason to suppose, that some undue effusion, or determination of blood in the brain existed, when he tells us, that his patient "although addicted to much talking in his health, yet his loquacity is now greatly increased." And few will dispute the probability of the same in the case I have detailed, from the fatuity and idiotic appearance conspicuous in the patient.

"Instead (says Dr. P.) of being solely a symptom of chronic hydrocephalus, chorea sometimes proceeds from obstructed obstructed menses, sometimes from exposure to rigorous weather, but generally from irritation in the first passages." I would ask, if the former of these supposed causes may not be itself reasonably supposed dependant on the disease of the brain? or if it be the original source of the disease, whether it may not act by the determination of blood, inducing effusion, &c. in the brain? In the same way may rigorous weather, and irritation of the first passages, promote chorea, by first inducing hydrocephalus.

As for convulsive agitations excited by particular irritations of the stomach; or choreatic gesticulations being attended by a spasmodic affection of the organs of deglutition; it would require more proof than the Doctor has brought, to convince me, that poisonous and other substances taken into the stomach, cannot excite determination to the brain, (as well as they can excite disorders of the skin) and thereby be a remote cause of convulsions; or, that the spasmodic affection of the organs of deglutition and the accompanying choreatic gesticulations, did not both depend on one common cause, viz. pressure on the brain.

The cases of Mr. Alexander, to which Dr. P. refers, are strongly corroborative of my sentiments respecting this disease, although Dr. P. has laboured hard to obviate the impression, which a review of the symptoms must necessarily create. In the first case, the symptom of strabismus occured, "a symptom (the Doctor says) of cerebral affection, yet hydrocephalus (he adds) shall not be the consequence." No indeed! it shall not be the consequence but was apparently the cause, both of it and of the chorea. The Doctor asserts that "the strabismus can be with reason ascribed only to the efforts of the brain to *recruit its impaired energy,* (risum teneatis!) but cannot, on good grounds, be imputed to hydrocephalus;" thus does theory oppose reason! The diminished tone of the alimentary canal, which the Doctor considers as the occasional cause of the convulsive motions, is certainly more rationally deduced from the pressure on the brain, to which it might then add additional force by the pressure of the contents of the alimentary canal on the descending aorta. "But surely, (adds the Doctor triumphantly) no theory, even the *newest,* can make us believe, whilst we are in our senses, (Q. E. D.) that every repetition of the strabismus was occasioned by the effusion of a fluid into some cavity of the brain, and that each cessation of the occular cular distortion was the effect of the absorption of that fluid." I will venture to ask if there is any thing remarkably difficult in this belief? Do we not see fluids effused and partially re-absorbed, and again recurring, in other cavities? And why is the brain alone to be an exception? If this belief is even not correct, it is not unreasonable to believe that the strabismus ceased, when the brain had become habituated to the pressure excited by the fluid, and continued absent, until by a fresh addition of fluid, an inordinate degree of pressure was again excited.

The "morbid condition of the alimentary canal" in Mr. Alexander's second case, in my mind, serves to strengthen the conviction, that the pressure on the brain was the cause of both it and the chorea: not, as the Doctor supposes, that this morbid condition "in a constitution of considerable mobility," (an expression conveying no precise meaning) is the general cause of the disease in question.

The third case, I view as equally conclusive on my side, (although Dr. P. gives it against me,) more especially as it serves to evince the propriety of a different treatment in different states of the disease. For that "the tonic and antispasmodic system of prescription" is *invariably* just, will I think be advocated but by few. Sydenham bled and purged repeatedly in this disease; and the medicines employed by Mr. Alexander, were best adapted to the stage at which they were employed, though they probably might not have proved equally serviceable in the commencement. I presume Dr. P. himself will acquiesce in the justice of this observation, and that he does not mean to say, that tonic and antispasmodic remedies are invariably proper.

The Doctor I observe, had some slight disposition to allow the doctrine to be just, (that chorea is a symptomatic disease,) when he adds, at the close of his remarks on Mr. Alexander's cases, that "they (the cases) all likewise tend to evince, that chorea is not a symptomatic ailment, *at least not naturally* symptomatic of hydrocephalus."— I must therefore believe that it has made more impression on the Doctor than he is willing to admit, or than his endeavours to overturn it, by his copious references, would seem to show.* The

* Both the symptoms and cure alike prove chorea to be *symptomatic* of pressure on the brain, and that it never is, nor never can be, an idiopathic disease. We might as well call the convulsive movements of hysteria, epilepsy, &c. idiopathic, without a reference to their primary source of which those movements are merely symptomatic!

The case brought forward from the Medical Repository, is one which depends on a cause, originally so different from any other, that it is truly difficult to reason upon it. I cannot however perceive that it adds any strength to the Doctor's ideas respecting chorea, nor that it precludes the possibility of the existence of water in the brain, either previously existing, or excited by the primary source of the complaint itself.

If Dr. Whytt has shewn, as Dr. P. asserts, "that a delicate state of the first passages, or a depraved sensibility of their nerves, not only disposes people to many complaints in those parts, but that the whole nervous system is thereby rendered more moveable, and liable to be affected by the slightest exciting causes;" may it not be equally inferred, that what is capable of exciting such morbid action in the nervous, is equally capable of producing similar results in the arterial system, and thereby of promoting effusion in various cavities of the body; in which the brain would be likely to be a principal sufferer?

Is it possible the case of Dr. Macbride, adverted to by Dr. P. could not awaken in his mind one idea, that in a disease of twelve years standing, the brain was enabled so to accommodate itself to the changes induced in it, that no symptoms of disease should be apparent, except of symptomatic chorea? Where is the Doctor's morbid condition of the alimentary canal, &c. &c. for so long a period? And why could not tonics and antispasmodics effect a cure? The answer is plain; because the chorea was not idiopathic, and the remedies were not adapted to the original disease.

The cases adduced from the Bristol Infirmary, are I think, strongly in my favour. The boy of 17, who received a blow on the head several years before, requiring the use of the trepan, although well till within three months of the attack of chorea, was apparently during that period, slowly receiving the impression from watery effusion, although this chronic state of hydrocephalus did not produce the symptom of chorea till so long after. The brain by slow effusion, conformed to the increasing pressure; as is surprisingly evident in every part of the body: and could the cases enumerated have been dissected, no one can dispute the great probability of finding the ventricles largely distended with water. In these instances, it can scarcely be called congestion; because the absorption of the brain would correspond with the slow deposition of fluid.

The

The girl who died of hydrocephalus, after being cured by zinc, shews a case similar to mine related. Here, for a time, by the use of zinc, chorea is suspended or cured; yet from some cause, an increased determination to the brain, (produced more suddenly than it had been accustomed to) converts the chronic hydrocephalus into its acute form, and with a fatal issue.

The other girl is cured by bark and the cold bath; remedies, apparently highly proper at that period, to excite the absorption of the existing fluid. Some analogy might here be drawn from the conversion of chronic rheumatism into acute, if it was necessary; but every one can apply the arguments which might be used.

As for the case of Dr. Hales, in the note, of a woman receiving a severe blow on the head, &c. succeeded by chorea, during the existence of which, no symptoms of hydrocephalus appeared, and in which the cure was effected by stimulants principally ;, I would ask, if such a blow was not likely to lay the foundation for effusion as the cause of chorea; although the brain may have accommodated itself to the slow deposition, and thereby have been uninfluenced by symptoms, which its more sudden effusion must have induced; especially as the Doctor allows that in a great majority of injuries to the head, no convulsions arise? And why? Because in general, in injuries of the head, venesection, the best preventive of morbid effusion from arterial action, is timely had recourse to.

The five cases collected by Mr. Mullen, in the Edinburgh Medical and Surgical Journal, which were under the care of that able and judicious physician Dr. Hamilton, I regard as highly instructive; inasmuch as they afford sufficient evidence of the great importance of constant purging in the complaint, which, so far from debilitating, added strength to the system, by probably exciting absorption in the brain; the influence of pressure on which, has a strong tendency to produce that apparently weakened but overburthened state of the system, (or what Dr. Brown has called indirect debility, and which is to be removed, not by tonics and antispasmodics, but by depletion. It is a great pity Dr. Patterson has not stated what Mr. M'Mullen has said of Dr. Brown, as it is very applicable to Dr. P. himself. "Dr. Brown," says Mr. M'Mullen, does not mention this disease particularly, but we can be at no loss for his sentiments with regard to it. With *his usual dogmatism*, he asserts that all spasmodic diseases are diseases of debility, and yield readily to tonics and antispasmodics. The first part of this assertion is hypothteical, and the second cond is erroneous." Here then we see Dr. P. has little to aid him, either in theory or practice, from the opinions of Mr. M'Mullen. And although I cannot agree with Mr. M'Mullen as to the cause of the complaint, I am truly rejoiced at the speedy triumph of the practice. And this the more so, because I see in its effects, the powerful, yet well known influence of evacuants, in promoting absorption of fluids in distant parts; and I see no one reason, on a careful perusal of the cases, to change my sentiments of the nature of the disease. Whatever may have been the cause of the black and fetid stools, there can be little doubt of the influence, which either their acrimony, or quantity, produced on the disease; for we find, that if at any time "the cathartics did not produce an evacuation, the involuntary motions recurred, and all the symptoms were aggravated." Now, that the suspension of the alvine discharge should augment the disease by checking the absorption of the fluid in the brain, or by giving an opportunity to the exhalants to throw out an additional quantity, is not surprising; and rather adds weight to the theory I have advanced. Be this as it may, I think I may confidently affirm, that if these cases give no sanction to my sentiments, they afford still less to those of Dr. Patterson.

The cases adduced by Mr. M'Mullen, of chorea produced by teething, and on which so great stress is laid by Dr. Patterson, do not I apprehend militate against me. It is true I may not be able satisfactorily to explain the effect this irritation might have induced on the brain, disposed, it is possible, (nay probable, as we so seldom see a like effect from the cause assigned,) to hydrocephalus: yet if Dr. P. will admit of analogy, I might mention the numerous cases of tetanic convulsions excited by a slight puncture in a very distant part of the body; and if such an effect may arise from a cause, apparently so trifling, it cannot be a matter of astonishment, that irritation of the gums may excite preternatural action in the vessels of the brain, and by causing effusion, induce a disposition to chorea; which a removal of the cause, (by preventing the increase or continuance of the effect, viz. effusion) would immediately obviate: One thing, however, is proved from the cases, viz. that however this irritation might act, the chorea induced was symptomatic, and not a primary disease: and this is confirmed by the case mentioned by Mr. M'Mullen from Dr. Gregory; in which two successive attacks of chorea were removed, by taking out the first set of teeth, by the side of which the second set were pushing up, up. At the age of 15, this boy was again attacked with chorea, when *no cause* of irritation was discovered *in his mouth*, and he was now cured with extract of cinchona and sulphate of iron. The important changes, which at the age of puberty take place in the system, may, I conceive, be a sufficient cause of increased action in the vessels of the brain, disposed, by former attacks on that organ, to effusion; which was removed by the tone and vigour given to the system generally, enabling the vessels of the brain in particular to resist the continuance of undue determination to them, or the absorbents to receive with equal facility, any fluid which the exhalants might pour into the ventricles.

That worms are very unjustly accused of exciting this disease by their irritation in the alimentary canal, I have endeavoured to shew in the essay which has called forth Dr. Patterson's remarks. That they may, when existing in a person affected with chorea, aggravate the disease, I have little doubt; but this must be by a primary effect induced on the vascular system, tending to augment the already overloaded vessels, or ventricles of the brain. The influence of purgatives in the cure of the cases related by Mr. M'Mullen, evince sufficiently, that when worms are in any case evacuated by purgative medicines, it is the operation of the cathartic, and not their mere expulsion, that benefits the patient; although, no doubt, much good may result from obviating a source of irritation, whether arising from worms or from a constipated state of the bowels.

Dr. P. has related the cases recorded by Sir George Baker, of certain poor children, "who by breathing vitiated air, were thrown into excessive tormina of the alimentary canal, which were attended with convulsions and delirium." Surely, without referring this to the indirect irritation on the *primæ viæ*, a nearer road might have led to the truth, when the *immediate* action of this vitiated air upon the lungs is taken into view; which, by not supplying sufficient oxygen to sanguify, (if I may use the expression without the lash of Dr. P's criticism) the blood, would produce such remora in the vessels of the brain, as may reasonably account for convulsions and delirium. Whether the tormina were produced by the vitiated air, or by (as is probable) unwholesome food; it appears evident, that both they and the convulsions were only symptomatic; and that the convulsions were less likely to be induced by the tormina, than by the cause I have assigned.

The

The three cases of chronic convulsions occurring in one family, as related by Dr. Armstrong in the ninth volume of the Edinburgh Medical Commentaries, and quoted by Dr. P. are so very extraordinary, as to leave little room for reasoning upon them. I see, however, small reason to connect them with chorea; and as I have undertaken to say, that *all* convulsive diseases depend on hydrocephalus, so I shall pass them over, without any further remark, than, that although no hydrocephalic symptom was apparent, it does not conclusively follow that that disease did not exist. Dr. Armstrong considered them as truly epileptic, and I see no reason for dissent.

As for what Dr. P. says, respecting general convulsions attending an herpetic eruption, or epileptic fits from the stimulus of bile, in rheumatic fever, &c. I have nothing to remark, as his observations are irrelevant to the subject in dispute; inasmuch as he is speaking of convulsions generally; and I am only contending for *one form of them*; and it would too far extend the limits of my paper, to wander into another field for argument.

"In genuine hydrocephalus internus, (says Dr. P.) convulsive affections do not occur, *until the disease be far advanced*, and the *choreatic species of those affections is the rarest stage of it.*" I think, was I disposed to attack the Doctor unfairly, I might say, this is an approximation to my opinion. I will however say, that this is the very thing I contend for. In the acute stage, the symptoms are rapid, and readily distinguished; in the chronic, *gradatim* and habit prevent the changes being so apparent.

"From a review, (adds Dr. P.) of 32 cases of genuine hydrocephalus internus, I find, that in 15 of them no convulsions took place; and that in any of the other 17 cases, spasms or convulsive motions did not occur, *until* the hydrocephalus had subsisted *several days*, nay in some, not until it had continued *several weeks*." "Besides, from a review of a few cases of spurious hydrocephalus, wherein the head was considerably enlarged, it appears that, in some of these, the convulsions *did not* attack *at the beginning*, and that, in others, even in those ascribed to external injuries, no convulsions appeared."

The last part of this paragraph, I add to prevent any idea of withholding evidence; though I have no where said, that either convulsions or chorea must inevitably follow the existence of hydrocephalus, but have distinctly asserted the contrary, without pretending to say, why it should so happen. As to the former part, I think it strongly strongly conclusive in my favour; as the acute hydrocephalus, strictly speaking, is rarely a disease of weeks; and the Doctor allows that the convulsions did not come on, till *long after* the presence of hydrocephalus! What more could I desire than this confession? for if the Doctor admits this, surely he might admit the same of chorea; which he above regards as a species of convulsions.

The cases adduced from Mr. Pott, to shew that in a majority of cases of wounds of the head, convulsive affections have not occurred, are entirely unconnected with our subject; for until the Doctor shews, that by convulsive affections he particularly means chorea, I cannot but think he is unnecessarily enlarging the original point of controversy. Yet even here I might safely meet him; for when particular information is given of the appearances on dissection of those who died, after convulsions had ensued, I think, without a reference to the cases, that suppuration, &c. producing pressure on the brain, was evident; evincing the convulsions to be the effect, (and consequently symptomatic) unless the Doctor can afford *stronger* grounds for belief that the convulsions caused the suppuration, than he has that chorea produced the effusion, evinced in the dissection of his and my cases.

As for Dr. P's assertion, that my styled "*new theory* is nothing more than that pathological doctrine, known since the days of Hippocrates, and termed *conversions* of *diseases*;" I can only say, I am sorry to see Dr. P. descend to subterfuges, to invalidate my claim to what I consider a just view of the doctrine of chorea. I think it needless to follow the Doctor in this part of the dispute; or to deny that any conversion of disease takes place in the complaint which is the subject of my remarks.* I considered the Doctor as a more open champion for his cause than I find him to be; and should therefore regard any triumph over him, (if the medical world should sanction my remarks) as less considerable, than I should be otherwise disposed to consider it. I shall, however, observe, that the Doctor appears to have brought cases even here, to support my theory; as, when he says "hydrocephalus internus has been transmuted into palsy, and palsy into hydrocephalus internus;" and furnishes us with, what he says, is an apposite instance of the latter, from Dr. Ferriar. "*Eight*

* If it be as old as Hippocrates, why has Dr. P. endeavoured to give it to Dr. Percival.

*months* after the appearance of the paralytic symptoms, the patient complained of severe head-ach; vision became indistinct, and at length was entirely lost. Epileptic fits *then* came on, and he died comatose. When the head was opened, the ventricles of the brain were found full of water, and several tumours, which in the prevailing medical language might be called scrophulous, were observed in different parts of the brain." Here it seems to me, cause and effect are very evident, without recurring to transmutation. The scrophulous tumours (as the patient laboured under them elsewhere) seem the original disease; and probably by their pressure induced the palsy. That they tended to excite the hydrocephalus, I have uo doubt; nor, that this hydrocephalus had been long advancing to its fatal termination; not as a chronic disease, but by some adventitious cause, evinced by the head-ach, &c. (the brain having accommodated itself by habit to its previously diseased and deranged state) which induced a sudden effusion, in addition, by which the symptoms of acute hydrocephalus became evident, and epileptic fits ensued. This also shews, that the epilepsy here was a symptomatic affection and not a primary one. It proves the connexion of those convulsive symptoms; and I will add, it strengthens the probable justice of my observations.

Of the conversions of disease, I have no doubt; but I must be permitted to question it, in the present instance. I would add the same of the case related from Dr. Percival, of pulmonary consumption converted into hydrocephalus, by the violent succussions of coughing. That the coughing induced the effusion of water in the brain, I doubt not; but I can only consider the absence of the cough, &c. &c. after the symptoms of hydrocephalus ensued, as a suspension of the one by the more violent action of the other. Still, however, if I was to admit all the Doctor's fallacious reasoning from the various cases he adduces, I do not think it would add one particle of strength to the weak fabric he has attempted to raise.

The case of Dr. P's patient, (neither "mutilated nor misconstrued by Dr. Coxe," as Dr. P. is pleased to affirm; but in which sentiment I trust no person will join him, after a candid perusal of the Doctor's own statement, and of the short abstract I have given of the case) is not, I venture to affirm, "a clear case of the conversion of chorea into hydrocephalus;" but in my opinion, demonstrative of the truth of the theory I have ventured to bring forward. In detailing detailing what I did of the case, it was not my intenton to criticise either the Doctor's opinions or practice, but merely to use those parts of it, which I imagined favourable to my sentiment. I shall here, however, analize more closely this case, in hopes of rendering those sentiments more plausible than in my former Essay;* and I think I shall make

---

* In this case (of a girl between eight and nine years of age) the patient had for some years "been liable to *frequent* tormenting head-achs, and *severe* stomach sickness; which were always removed at the time by an *emetic*, whose operation pumped up some bile." A few weeks before Dr. Patterson's visit, (Ap. 3d, 1790) "an unsteadiness of her limbs' was first observed, with other symptoms of chorea. "She had a catarrhal complaint attended with a *severe cough*, before the convulsive gesticulations were noticed." The Doctor found her pulse *slow* and small; and the eyes regular and sound. Bowels tardy, their habitual disposition. Head free of pain, and disturbance, &c.: a blister was applied between the shoulders; a flannel shift ordered; and the most acceptable diet to her palate.

I would here ask, whether the long continued head-ach to which she was subject, may not be imputable (if not to the deposition of water, at least) to an augmented determination of blood to the brain, at those periods of the accession of head-ach? and whether the severe stomach sickness, and use of emetics, might not have been continually increasing the disposition to effusion, by still further adding to the determination of blood, in a part apparently in a weakened state? I would ask likewise, whether the severe cough before the convulsive gesticulations were observed might not have produced the same effect, as is allowed to have occurred, not however by conversion of disease, in Dr. Percival's case, as noticed in Dr. Patterson's observations, with the view of shewing the influence of coughing upon the brain? Here then we have some room, at the very commencement of the history of the case, to apprehend the existence of the effusion, which Dr. Patterson supposes was the *effect* of the chorea; but which appears to me, more properly to be regarded as the effect of pressure, then existing.

It is by no means requisite to notice the daily prescriptions, which were chiefly of the tonic and antispasmodic kind, accompanied with occasional purgatives, exercise, the warm and cold bath, electricity, &c. I shall therefore observe, that on the 10th, after apparently mending, "an alarming faintishness came on, succeeded by languor, oppression, and much agitation," for which ether and laudanum were given. On the 23d, a little head-ach, for the fourth time since her present illness. She vomited twice on the 22d. On the second of May, she began with zinc and columbo. On the fourth, her hair was cut short, and her head dipped in the cold bath; her appetite diminished, yet her strength was as good as before.

15th. Sickness and vomiting, &c. 21st. Removed into the country; in every respect seemed gaining ground. From this date to the 21st June, she was not seen by Dr. P. but regular accounts were transmitted to him.

24th. Fatigued by ride. Very sick, retched, vomited, and threw up phlegm. 26th. Agitations morning and evening.

27th. So much agitated that the antispasmodic medicine was given along with the tonic. She has some little use of the *left*, but not any of the *right*, hand.

make it appear, that it was truly a case of symptomatic chorea, arising from the pressure of water in the brain, which

28th. Very sick. Little or no use or power of the right hand; the left she can do almost any thing with.

I shall here beg permission to inquire on what could depend the total loss of power of the *right*, whilst the *left* hand remained capable of doing almost any thing? Surely we shall not be told that the chorea was the cause! I will ask, what more reasonable explanation can be given, than that pressure, and that partial, on the brain, produced this effect? and if pressure, what more likely than that it arose from water, already existing in the ventricles; which the dissection but a short time subsequently, proved to amount to three ounces? It has been observed, that injuries sustained on one side of the head, produce a paralysis of the opposite. Now, as the dissection shews that two ounces of fluid existed in the *left* ventricle, and only half that quantity in the *right*, may we not imagine it to induce this paralysis most particularly on the right-side?

June 8th. Sick again. I must observe that my reasons for mentioning the periods of her sickness at stomach, which was accompanied with vomitting, is, that I consider the straining induced thereby, as adding greatly to the determination of blood to the brain, and consequently rendering the disposition to effusions still greater.

It is subsequently observed, that "notwithstanding these two sick days she had got strong and steady in the muscular powers, so much so that she was able to run about, to use her left hand nearly as well as ever, and to command her right hand in a visibly progressive degree of improvement." This I should apprehend, depended on some degree of absorption of the effused fluid, by which the pressure on the brain was diminished. This state of improvement was however but short, for on the 13th she had much head-ach and sickness of stomach, which continued without relief three days: and on the fourth, by domestic advice, she got two grains of tartarized antimony, with camomile infusion, which excited a watery evacuation by vomiting; her night's sleep was worse than usual; appetite much impaired; involuntary motion of the right hand; and what she looked at appeared clouded, or rather striped with red and purple. Do not these symptoms indicate increased determination to the brain? And what so likely to increase it as the emetic?

18th. Head and sickness so bad that a second emetic was given, with the discharge of much bile and phlegm; and seemingly with relief; but the head-ach next morning was equally bad. The bath was used, and being as ill as ever, she begged for more tartarized antimony, and went to bed very feverish. About nine that evening three grains of James's powder were given, with no visible effect. She passed a tolerable night, but soon after waking, her head was as bad as previously.

20th. Four grains of the fever powder at eight in the morning, which proved *emetic*. Slept a good deal in the course of the day,* yet complained much

* In fpeaking of a cafe attended by Dr. Quin in 1771, (at p. 5, 6,) Dr. Patterfon fays, "for we do not find the idea held by the parents, namely, that of the difeafe being a common fever, was contradicted by the Doctor, who accordingly permitted the adminiftration of *James's* powder; the confequence of which was, *as might be expected*, that the ftupor very foon came on." Now, can we fuppofe, that in Dr. Patt rfon's cafe, the ufe both of James's powder and emetics, would be lefs likely to p oduce a fimilar refult?

which the present explanation of Dr. P. serves but to confirm, although he affirms it to come under the second sub-division,

---

much of her head; powder repeated at five and ten in the evening; a gentle perspiration of some hours. Head-ach, quick pulse and heat undiminished; the heart beat remarkably rapid; much flushed. Could not water be yet suspected?

21st. Very little sleep; very hot, pulse quick, but not so strong as yesterday; sighs frequently; purgatives produced no effect.

22d. Dr. Patterson saw her; and found her complaining *grievously* of the *head-ach*; fits of incoherent muttering, but in general sensible; face *flushed*, eyes composed, and regularly affected by light, &c.; pulse 90, hard and full.

From this particular attention to the state of the eyes, then, it is probable the Doctor now began to regard it as a case of hydrocephalus, but not finding the symptoms he expected, he cannot yet view it as such; and we must conclude, his mind did not embrace the idea of water in the ventricles, until the coma and strabismus came on, on the 25th.

"Convulsive agitations vanished; and is possessed of as full power of her hands, &c. as consistent with her lately diminished strength." Blisters to the head, effervescing mixture and infusion of columbo freely given; emollient injections; two grains of calomel every night; and a few drops of the baic tincture were added to two doses of the neutral mixture, to compose the anxiety and restlessness.

23d. Rested tolerably; blister operated fully; much relief of head; pulse and other circumstances as yesterday; *vomited* soon after dressing the blister, &c. The Doctor returned home, and was informed by letter the next day, that soon after leaving her, "she was seized with a violent delirium, raving incessantly, &c." An emollient injection produced an evacuation but no relief; she *lost her speech suddenly*, and groaned continually. &c. In some time a copious sweat broke out. It appeared she had suffered a paralytic stroke, as evinced by a distortion of the mouth, and a subsequent privation of her right hand. *Quere?* What did the previous privation of her right hand evince?

Here, I may observe, that the *chronic* hydrocephalus appears to be converted into the *acute* state, by some additional effusion, more rapidly made, than the brain could conform to; which I attribute to the action of the repeated emetics; for now, 25th, the Doctor found her in a *comatose* state; low delirium; *strabismus* towards the left side; *pupils dilated* and *insensible* to light: flushed; breathing quick; suspiria; partial perspirations; convulsively affected in the *left* side; *torpid* in the *right* side; deglutition much impaired; pulse 110, and unequal; costive.

26th. Quiet during the night; but coma, &c. continued; *left* arm and hand more convulsed; *right* arm continues motionless.

This shews the affinity of the paralysis, and the convulsions; and that both were symptomatic, arising probably from pressure on different portions of the brain, and differing in degree.

27th. General convulsions last night, between 11 and 12, recurring at different intervals, and of various strength until the last; death closing the scene about two the ensuing morning.

 DISSECTION.

division, third head of conversions, thus laid down by D Ferriar. "If th original be a chronic disorder, such a state of the hab t (f vourable t the production of another disease), may take place during its continuance, an l the accessory disease may be simply super-added, or it may vary the form, or affect the duration of the former."

The case I have first related, " which (says Dr. P.) inspired him (Dr. C.) with the rudiments of the *new theory*, was a case of similar conversion," I can only say that this conversion (or perversion) exists only in the Doctor's imagination. It was this case, and the dissection, which awakened

---

DISSECTION.

Blood vessels of dura mater *turgid* and reddish, exhibiting strong marks of a preceding inflamed state. About two oz. of blood flowed from the longitudinal sinus by wounding it with the saw. Cortical and medullary substance of brain shewed no other morbid appearance than a kind of *serous diffusion* over the circumvolutions of the lobes: the left lateral portion of the plexus choroides seemed inflamed. From the left ventricle two ounces of watery fluid issued, and half that amount from the right. No other signs of disease observed.

I have thus, without giving the minute detail of each day, stated the various circumstances which appear to lead inevitably to the conclusion I have drawn; and I believe it is unnecessary to enlarge my remarks to a greater extent, as the whole of the Essay will give my explanation of the various symptoms After reading this case, I think few can doubt that the convulsions and the paralysis, were here the symptomatic effects of pressure from the water; for it is not to be credited, that so much water could have been evacuated in so short a time after the symptoms of hydrocephalus enumerated on the 25th, and the decease of the patient on the 27 h I must therefore still believe, that every circumstance upholds the truth of the opinion I have advanced, that the chorea, &c. were induced by the w,ter which had been gradually accumulating, probably from the o i nal commencement of her head-ach, many years before; and that the effects of the emetics on the 17th and 18th of the month, were, by inducing too great a determination to the brain, to induce the inflammation apparent, and turgescence of the vessels, and thereby, also, probably to cause an additional effusion, inducing coma, delirium, strabismus, and dilated pupils, &c.

I shall not follow the Doctor in the observations which the case has led him into; except to say, that I think his case proves, that the ancients and most of the moderns" were right in considering chorea under the general head of convulsions; " although it be attended with sev ral peculiar circumstances." I would ask, what but these " peculiar circumstances," could discriminate it from other species? Cullen then, has not corrected an error, but has fallen into one, when he acknowledges, in the later editions of his Synopsis, that he was mistaken in supposing it to be merely a variety of those maladies. His stumbling from truth into an error, is greatly to be regretted, since even the errors of so great a man are so readily embraced.

awakened in me a desire of investigating the subject, which till then had not been particularly the object of my attention. Will the Doctor affirm, that the chorea was converted into hydrocephalus, when chorea existed to the last; or that so large an accumulation of water as 12 oz. could have taken place suddenly, without producing instant death, when the slightest attention to the circumstances of the dissection must evince, that it must have been slowly accumulating? The brain, incapable of yielding, must have gradually accommodated itself by its absorption. I can now readily see the reason why I did not then suspect hydrocephalus; for I had no idea that the chronic hydrocephalus could progress, with so few characteristics of its acute form. I was, indeed, then in the dark, and should truly have taken in "good part" the Doctor's endeavours (however feeble) to furnish me "with some *light* upon the subject." His case I thank him for, as it has done more than his arguments; but to be led by him, I should regard (if I am wrong) as the case of the blind leading the blind; and the event which would follow, the Doctor well knows; I hope, therefore, he will "take in good part" my leaving him to grope by himself, unless these remarks may aid him; as I conceive his doctrines require much further elucidation than he has thrown upon them.

If the Doctor had read with candour my explanation, I think it impossible he would have thus expressed himself. "Very much in the dark, indeed, must he have been, and labouring under great perplexity, when we find him expressing his ideas in the following confused and contradictory terms." 'The *water* must have been accumulated (*accumulating*, I apprehend it must have been in the original) a *considerable time*, as evinced by the quantity, and the very enlarged state of the foramen ovale. Its *sudden effusion* must have produced apoplexy, but the *slow progress* of the effusion permitted the brain to accommodate itself to the pressure.' 'The *water in the brain* was not, I believe, the immediate cause of death. Some *sudden effusion* or congestion, was the source of the fatal issue, by producing apoplexy.'

Few persons will consider the first quotation of mine, given by the Doctor, as confused or contradictory, and I apprehend the deduction at the end will be opposed by yet fewer; a due attention to the text and context might have shewn the Doctor, that the last quotation does not contradict the former; for, I apprehend, it will be allowed, if the brain had accommodated itself to this great

 pressure, pressure, it could not be the immediate cause of death in itself; and yet that some sudden effusion or congestion in addition, above its capability of accommodation, might very probably induce apoplexy and death.

I shall not say much on the illiberal and ungentlemanly attack on the "*medical treatment*," as it would savour too strongly of the *disposition* which led Dr. P. to employ his pen so *unworthily* upon a supposed intention to offend him. I shall merely say, that I candidly stated it (whether right or wrong); as I considered it proper in investigating the subject, to withhold no circumstance, even if militating against me. I think, however, that whoever reviews the immense proportion of fat in all parts (as shewn by the dissection), the inordinate appetite, and the enormous accumulation of blood in the vessels of the head, will see reason to dissent from the idea of Dr. P. of the patient being in a "reduced condition," although he had lost above 100 ounces of blood (in above four months, a quantity by no means uncommon in as many days, on this side the Atlantic, and which might probably have *really* benefited the patient under consideration, if he had lost it in the same space of time). If the Doctor has no more sense of the propriety of conduct due from one gentleman to another, and especially of that decorum, so essential in members of the same professson; I would at least advise him to review his own practice, and see if the unwarrantable application of the line addressed to me, may not reverberate upon himself.

( To be continued. )

### *To the Editors of the Medical and Physical Journal.*

GENTLEMEN,

